# Epilepsy: unusual presentation of cerebral hydatid disease in Children

**DOI:** 10.11604/pamj.2016.25.58.10706

**Published:** 2016-10-03

**Authors:** Farouk Hajhouji, Khalid Aniba, Mehdi Laghmari, Mohammed Lmejjati, Houssine Ghannane, Said Ait Benali

**Affiliations:** 1 Department of Neurosurgery, Mohammed the sixth University Hospital, Marrakesh, Morocco

**Keywords:** Cerebral hydatid disease, echinococcosis, CT, MRI, surgery

## Abstract

Cerebral hydatid disease is very rare, representing only 2% of all cerebral space occupying lesions even in the countries where the disease is endemic. Intracranial hydatid cysts are more common in children and occur more frequently in the supratentorial space. The aim of this paper is to describe the characteristic features of computed tomography (CT) and magnetic resonance imaging (MRI), and to determine the clinical presentation and surgical outcome of cerebral hydatid disease. A 7-year-old girl was admitted to the emergency department because of an epileptic attack. On radiological examination a round, cystic lesion appeared in the parietal lobe and caused shift of the midline structures. The cyst was successfully removed using the dowling technique. The postoperative period was uneventful and seizures were not seen during follow up. Hydatid cyst of the brain presents clinically as intracranial space occupying lesion and is more common in children, it is well demonstrated by CT and MR examinations, and Surgery is the treatment option with affordable morbidity and low mortality.

## Introduction

Hydatid disease (echinococcosis) is a worldwide zoonosis produced by the larval stage of the Echinococcus tapeworm [1]. This is an endemic manifestation that is more frequently found in Mediterranean countries, the Middle East, South America, and Australia, and this entity affects particularly those involved with sheep and cattle rising [2]. However, because of modern day travel and population movement, it may be encountered in any part of the globe [3]. It is important to be aware of the condition even in nonendemic parts of the world, where only occasional cases are encountered. Cerebral hydatid cysts are extremely rare, forming only 2% of all intracranial space-occupying lesions [4]. Clinically, symptoms and neurological signs depend on the size and location of the cyst [5]. The diagnosis relies on serologic tests and imaging techniques CT and MRI. Because of his rarity, experience with intracranial hydatid disease at a single institution has been very limited, and the Dowling technique is widely used as a surgical treatment [2]. In this report we describe the clinical features, radiological findings and surgical outcome of cerebral hydatid cyst. We also review the relevant literature.

## Patient and observation

**Figure 1:**
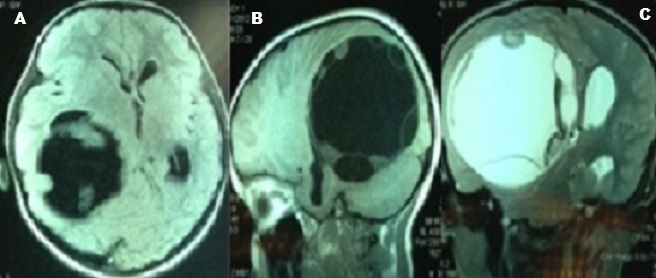
A) axial T1 weighted MR image shows a large hypointense parietal cystic lesion; B) sagittal T1 weighted MR image shows daughter cysts; C) coronal T2 weighted MR image shows mass effect and shift of the midline structures

*History and examination:* This 7-year-old girl, previously healthy, was admitted to the emergency department for an epileptic attack, she described four episodes of partial seizure secondary generalized. Neurological examination revealed postictal confusion, hemiparesis and bilateral papilledema. Headache and vomiting were not experienced. An anticonvulsant therapy was administrated. Magnetic resonance images (MRI) demonstrated well-defined round (spherical) entirely surrounded by brain parenchyma hypointense lesions on T1-weighted images (Figure 1 (A,B)) and hyperintense lesions on T2-weighted images (Figure 1 C). The walls of the cyst were hypointense on T1- and T2-weighted images (Figure 1) and contain few peripheral daughter cysts. No calcification, no surrounding edema, and no contrast enhancement were seen. Preoperative radiological diagnosis of cerebral hydatid cyst was suggested. Abdominal ultrasonography and X-ray chest were performed and failed to reveal any associated hydatid cyst in lungs and liver.

**Figure 2:**
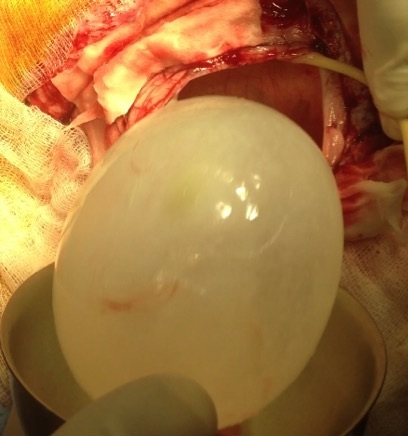
Large hydatid cyst delivered without rupture

*Operation:* emergency surgical intervention was performed. After craniotomy the entire cyst was removed without rupture during the dowling technique (Figure 2). Histopathological examination confirmed the diagnosis of hydatid cyst.

**Figure 3:**
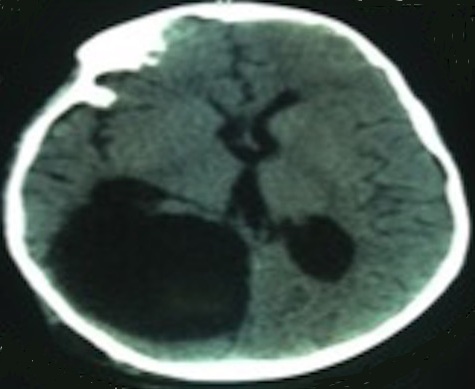
Postoperative CT shows total removal of the lesion

*Postoperative course:* the patient’s postoperative course was uneventful; the patient’s neurological status remained good. Postoperative CT confirmed total removal of the lesion (Figure 3) she was discharged from the hospital on postoperative Day 5. Full recovery took place one month later. Seizure or recurrences were not seen during follow-up period.

## Discussion

Hydatid disease, although rare, is usually seen in endemic areas of sheep-raising countries. Humans acquire the disease mostly during childhood. The liver (50-77%) and the lung (8.5-43%) are the organs most commonly involved. The remaining lesions may involve any organ in the body, including cerebral involvement in approximately 2% of patients infected with the parasite [1]. They may reach a considerable size before the patient becomes symptomatic. There is no consensus on the growth rate of the hydatid cyst of the brain and has been variably reported between 1.5–10 cm per year [6].

In cystic echinococcosis, the parasitic cyst consists of an inner germinal layer (endocyst) and an outer laminated layer (ectocyst). The host reacts to the cyst by forming a fibrous capsule (pericyst), which contains blood vessels that provide nutrients for the parasite. From the germinal layer, scolices, brood capsules, and daughter cysts are formed by endoproliferation (internal budding) [1].

Cerebral cystic echinococcosis is most commonly seen in children and young adults (approximately 50–70%) [1]. El- Shamam et al. [7] and Lunardi et al. [8], reported that cerebral hydatid cysts are commonly seen in children especially in males and young adults. Beskonaklí et al. [9] reported a reasonable explanation for this, as young male children were more closely occupied with animals than girls or adults and were not as aware of the importance of hygienic principles.

The lesions may remain asymptomatic until they are quite large. Headache and vomiting were the most commonly reported symptoms in other series. Other symptoms such as hemiparesis, seizures, visual field alteration and gait disorders, may vary with the location of the cyst [7]. Papilledema is usually present in patients with intracranial hydatid cysts at the time of diagnosis [8].

Lesions of cerebral hydatid disease are usually distributed in the territory of the middle cerebral artery, especially in the parietal lobe. Most of the cysts are located in the supratentorial regions [1, 6], and very rarely in the posterior cranial fossa, or ventricles [1, 5].

Multiple cerebral hydatid cysts are rather rare and result from spontaneous, traumatic or surgical rupture of a solitary primary cyst or as a consequence of a cyst rupture elsewhere and embolization of hydatid to the brain [1, 10]. These secondary cysts, which are infertile, do not have a thick capsule [10].

Patients with cerebral hydatid cysts may have hydatid cysts in other organs. In recent studies, hepatic, pulmonary and other locations were found in 10% of the cases [11]. In the series of Ciurea et al. [12], 8 patients, presented hydatid infestation in multiple organs. El-Shamam et al. [7] reported only one patient with involvement of other organs out of 16 patients.

Both CT and MRI demonstrate a spherical and well-defined, smooth, thin walled, homogeneous cystic lesion with fluid density similar to the cerebrospinal fluid, with or without septations or calcification. On unenhanced CT, the cyst wall was isodense or hyperdence to brain tissue. The cyst wall usually showed a rim of low signal intensity on both T1- and T2-weighted images. Calcification of the wall was rare, being less than 1%. The presence of daughter cysts is considered pathognomonic but has been rarely reported [1]. Compression of the midline structures and ventricles are seen in most of the cases, however surrounding edema and rim enhancement were usually absent in untreated or uncomplicated cases [1, 4]. Obvious mass effect was demonstrated.

The differential diagnosis of intracerebral hydatid cysts includes cystic lesions such as porencephalic cyst, arachnoid cyst, cystic tumor of the brain and pyogenic abscess. In contrast to hydatid cysts, porencephalic cyst and arachnoid cysts are not spherical in shape and not surrounded entirely by brain substance. Arachnoid cysts are extra-axial masses that may deform adjacent brain. Porencephalic cysts result from insults to normal brain tissue and are lined by gliotic white matter that could easily be demonstrated with MR imaging. Cystic tumors of the brain could be differentiated by the enhancement of the mural nodule, if any, and periphery of the tumor. When a pyogenic abscess shows a cyst-like central necrotic area; peripheral edema is almost always present, the rim enhances intensely following contrast administration and satellite lesions are commonly present, all of which could be demonstrated on both CT and MR images. Clinical and laboratory findings could also aid in the differential diagnosis of the patients with cerebral abscess formation.

The diagnosis should be suggested by evidence of a primary hepatic focus, appropriate clinical history, high prevalence of the infection in the host’s geographic location, and laboratory findings [1]. Cerebral cysticercosis should also be kept in mind in the differential diagnosis of cerebral echinococcosis. According to the literature, in a small number of cases diagnosis was established preoperatively on CT and MRI [1]. Serologic analysis is in most cases negative; pathohistologic analysis is the most reliable method of diagnosis. Therefore, cerebral cysticercosis or echinococcosis should be suspected especially when cystic lesions appear in the central nervous system [13].

In conclusion, CT and MR imaging, alone or in combination, are helpful in the diagnosis of cerebral hydatid disease. Although CT is superior in detecting calcification of the cyst wall or septa, when present, MR is better in detecting multiplicity and defining the anatomic relationship of the lesion with the adjacent structures and helps in surgical planning. MR provides additional information and details that cannot be seen by CT. It provides additional information in the exact localisation of the cyst. When present, in complicated or recurrent disease, surrounding edema can better be demonstrated with MR imaging owing to the inherent capability of the imaging modality in revealing subtle differences in the tissue content. When a well-defined spherical cystic intracerebral lesion with obvious mass effect, but no surrounding edema and no contrast enhancement following contrast administration is detected on CT and MRI, hydatid disease should be taken into consideration in countries where the disease is endemic. If perilesional edema and thin rim enhancement are observed either complicated hydatid cyst or other cystic lesions of the brain should be considered in the differential diagnosis.

The treatment of cerebral hydatid cysts is principally surgical. The primary goal of the operation is total cyst extirpation without rupture to prevent anaphylactic reaction and local recurrence [12]. Many different techniques of cyst removal have been proposed and all of them emphasize atraumatic techniques to avoid cyst rupture. The Dowling technique later improved by Arana-Iniguez and San Julian [14], has been widely used for the surgical treatment of hydatid cysts of the central nervous system. The essential steps of this technique are the following: creation of a large flap; careful handing during all operative steps to avoid monopolar coagulation; opening the atrophic cortex overlying the cyst over an area with a diameter no less than three quarters of the diameter of the cyst; and allowing the cyst to fall out by just lowering the head of the operating table and flushing warm saline between the cyst and surrounding brain [2].

Despite the advancements in microsurgical operative techniques and instrumentation, cerebral hydatid cysts pose a challenge for the surgeon because of the following characteristics: they are usually diagnosed when they are large in size; they have a very thin cyst wall; the neurological deficits are often minimal in their presentation despite the location and the large size of the cyst; and they are sometimes located deep or near the ventricular wall and require retraction of vital structures or meticulous cortical dissection. There are many reports suggesting that the Dowling technique is the most effective surgical procedure for the removal of cerebral hydatid cysts. However, some pitfalls exist with this technique concerning surgical methods, instruments, and cyst location. The best operative approach to a cystic lesion in the brain should be based on the site and the size of the cyst, and the relationship of the lesion with the other neural and vascular structures.

Few reports mention the efficacy of drug therapy with albendazole in the cure of cerebral hydatid cyst [3]. However drug therapy is better combined with surgery in case of accidental cyst rupture.

## Conclusion

We concluded that hydatid disease is not uncommon, affects pediatric age group and is usually located supratentorially. The clinical features are those of intracranial space occupying lesion. It can safely be removed intact with the dowling technique.
